# COVID Perceptions among Pregnant Women Living in a Malaria Hyperendemic Rural Region in Uganda: A Cross-Sectional Study

**DOI:** 10.4269/ajtmh.23-0464

**Published:** 2023-11-06

**Authors:** Francesco Vladimiro Segala, Giulia Patti, Lameck Olal, Elda De Vita, Nelson Olung, Roberta Papagni, James Amone, Valentina Totaro, Emmanuel Onapa, Roberta Novara, Benedict Ngole, Mariangela L’Episcopia, Samuel Okori, Giovanni Dall’Oglio, Jerry Ictho, Carlo Severini, Giovanni Putoto, Peter Lochoro, Francesco Di Gennaro, Annalisa Saracino

**Affiliations:** ^1^ Clinic of Infectious Diseases, Department of Precision and Regenerative Medicine and Ionian Area, University of Bari “Aldo Moro,” Bari, Italy;; ^2^ African Network for Change, Kampala, Uganda;; ^3^ St. John’s XXIII Hospital Aber, Jaber, Uganda;; ^4^ Department of Infectious Diseases, Istituto Superiore di Sanità, Rome, Italy;; ^5^ Doctors with Africa, CUAMM, Kampala, Uganda;; ^6^ Operational Research Unit, Doctors with Africa CUAMM, Padua, Italy

## Abstract

Both SARS-CoV2 and *Plasmodium falciparum* infection during pregnancy increases the risk for adverse maternal and fetal outcomes, including abortion, severe disease, and death. Indeed, although malaria and COVID-19 show an overlapping clinical presentation, they require a profoundly different approach. The aim of this study was to explore COVID-19 awareness among pregnant women living in a *P. falciparum* hyperendemic region in rural Uganda. This cross-sectional, prospective study was conducted in one Hospital and two Health Centers (HC) in Lango region, Uganda, from July 14, 2022, to March 14, 2023. Data about demographics, COVID-19 history, and COVID-19 and malaria perceptions were collected using RedCap mobile app platform. Study endpoint was a context-specific COVID-19 awareness score, accounting for the most common disease misconceptions. Association between study variables and good COVID-19 awareness was assessed by χ^2^ and *t* test, as appropriate, and variables found to be statistically significant were further explored in multivariate logistic regression analysis. A total of 888 pregnant women were recruited. Median age was 24 (interquartile range: 20–29) years, whereas 79% (*n =* 704) attained only primary education and 66.6% (*n =* 591) were used in agriculture. SARS-CoV2 vaccination rate was 92%. In multivariate analysis (Table 3), variables associated with high COVID knowledge were presenting at antenatal care visit in Atipe HC (adjusted odds ratio [aOR]: 8.1, 95% CI: 4.1–16.48) having a previous good knowledge about malaria (aOR: 1.76, 95% CI: 1.21–2.56). Among pregnant women living in rural Uganda, COVID-19 awareness relies on the overall educational level, malaria knowledge and reference HC. Among pregnant women living in *P. falciparum* endemic areas, community-level malaria awareness might guide educational interventions during future pandemics.

## INTRODUCTION

In 2020, the WHO reported an increase in the estimated total number of malaria cases occurring globally, reaching 247 million cases from the 230 million reported in 2015. The rise that occurred in 2020 has been largely attributed to the disruption of malaria control strategies caused by COVID-19,^1^ because malaria control relies on individual choice to seek care, while people were warned to stay home in case of fever; supplies such as insecticide-treated nets or antimalarial drugs could not be provided; and the healthcare workforce was constrained.^2^

In this context, pregnant women are particularly vulnerable because of their increased risk of developing severe malaria and of experiencing preterm delivery and intrauterine growth retardation due to a buildup of* Plasmodium falciparum*–infected red blood cells in the placenta. In malaria hyperendemic regions, pregnancy is a risk factor for both the acquisition of *P. falciparum* infection and the development of severe malaria. Complications of malaria infection in pregnancy affect both the mother and the child and include maternal anemia, low birth weight, prematurity, infant malaria, childhood anemia, and congenital malaria.^3^ Likewise, compared with nonpregnant women of reproductive age, pregnant women infected by SARS-CoV-2 are at increased risk of mortality and of progression toward severe disease requiring respiratory support, admission to an intensive care unit, invasive ventilation, dialysis, or extracorporeal membrane oxygenation,^4^ also due to the still poor therapeutic options available, which makes them more vulnerable to the progression of the disease. Furthermore, babies born to mothers with COVID-19 were more likely to be admitted to the neonatal intensive care unit than those delivered to non-COVID mothers.

As a consequence, during the COVID-19 pandemic, pregnant women living in communities with high-malaria burden had to face the multiple challenges posed by two highly incident diseases that had similar clinical presentation,^5^ threatened their own life and the life of their fetuses, but required profoundly different approaches in terms of community prevention, healthcare seeking, and disease control.^6^^,^^7^ Also, for both diseases, community-level awareness demonstrated to be among the main driver of prevention success.^8^^,^^9^

In Uganda, the country with the third highest burden of malaria cases worldwide,^1^ COVID-19 cases progressively increased with three major waves that peaked in December 2020, June 2021, and January 2022, respectively, leading to a total of nearly 172,000 cases and 3,632 deaths up to August 2023.^10^^,^^11^ Furthermore, Oyam district, a rural area in Northern Uganda, has a malaria incidence and mortality both above the national average.^12^ In Lango region, *P. falciparum* is responsible for 97% on malaria cases, and overall maternal mortality is 336 maternal deaths every 100,000 live births.^13^ However, despite the high vulnerability of pregnant women to both *P. falciparum* and SARS-CoV-2 infection, little is known about the interplay between these two conditions in terms of disease awareness, behaviors, and preventive attitudes.

The aim of this study was to explore awareness of COVID-19 among pregnant women living in a *P. falciparum* hyperendemic region in rural Uganda.

## MATERIALS AND METHODS

This study is a part of a broader research project “Impact of Antimalarial Resistance and COVID-19 Pandemic on Malaria Care among Pregnant Women in Northern Uganda (ERASE).”^2^

### Study design, setting, and population.

This is a cross-sectional, prospective observational study. The study was conducted in one Hospital (St John’s XXIII Hospital of Aber) and in a third and fourth level Health Centers (HCs)^14^ (Atipe HC and Aboke HC), located in the districts of Oyam and Kole, Lango Region, Uganda. All pregnant women presenting to the antenatal care clinic between July 14, 2022, and March 14, 2023 were eligible for recruitment. Study protocol was approved by Lacor Hospital Research and Ethics Committee (prot. no. LACOR-2022-95). All women included in the study provided written informed consent.

### Study procedures.

Data were collected by administering structured questionnaires. Study questionnaires were prepared together with local health operators and community leaders. Malaria knowledge was evaluated with a 5-point questionnaire based on the Malaria Indicator Survey developed by Ugandan Ministry of Health.^11^ From June to August 2020, a dedicated training about COVID transmission and prevention measures was carried out in all study centers informing the population about local epidemiology of COVID-19, SARS-CoV2 route of transmission, risk factors for pregnant women, and prevention methods such as use of facemasks and adherence to the vaccination campaign. Study data were collected and managed using REDCap Mobile App electronic data capture tool^15^ hosted at Catholic University of the Sacred Heart, Rome.

### Study endpoint.

Endpoint of the study was a context-specific, internally validated COVID-19 awareness score developed with local community leaders and healthcare operators. The awareness score accounted for the most common disease misconceptions in Oyam and Kole districts. The score ranged from a minimum of 0 and a maximum of 5 points.

### Statistical analysis.

A descriptive analysis was performed to define the distribution of baseline variables and characteristics of the sample. The dependent variable was COVID-19 awareness, and a score of at least 4 points was considered as “good knowledge” of COVID-19. Continuous variables were compared between groups using independent *t* tests, and categorical variables were analyzed using a χ^2^ test. A logistic regression model was used, with COVID-19 good knowledge as the dependent variable and the available factors at the baseline evaluation as independent variables in the univariate analysis. Factors with a *P* value < 0.10 from the univariate analysis were included in the multivariate analysis. The strength of the association between baseline factors (exposure) and COVID-19 knowledge (outcome) was measured using adjusted odds ratios (aORs) with 95% CIs. All statistical tests were two-tailed, and a *P* value < 0.05 was considered statistically significant.

Statistical analyses were performed using R Statistical Software (v4.1.3; R Core Team 2021) in R Studio Version.^15^

## RESULTS

As shown in Table 1, a total of 888 women were recruited in the study. Almost all (*n =* 883; 99.4%) lived in rural setting. Seven-hundred and nineteen women (81%) were recruited in Aber Hospital, and 97 (10.9%) in Aboke HC and 72 (8.1%) in Atipe HC. Median age was 24 (interquartile range: 20–29) years. Seventy-nine percent of the population (*n =* 704) attended only primary school, whereas only 4.7% (*n =* 42) reported to have attained tertiary education. Most of the women were farmworkers (66.6%, *n =* 591). Ninety-three percent (*n =* 830) reported being married.

**Table 1:** Descriptive statistics and monovariate analysis

Variable	Overall (*N* = 888)	Low COVID awareness (*n* = 715)	High COVID awareness (*n* = 173)	*P* value*
Age at enrollment				0.57
Median (IQR)	24.0 (20.0–29.0)	24.0 (20.0–29.0)	25.0 (21.0–29.0)	
Missing, *n* (%)	2 (0.2)	2 (0.3)	0 (0)	
Setting, *n* (%)				0.839
Rural	883 (99.4)	713 (99.7)	170 (98.3)	
Urban	2 (0.2)	1 (0.1)	1 (0.6)	
Missing	3 (0.3)	1 (0.1)	2 (1.2)	
Health center, *n* (%)				**< 0.001**
Aber	719 (81.0)	604 (84.5)	115 (66.5)	
Aboke	97 (10.9)	78 (10.9)	19 (11)	
Atipe	72 (8.1)	33 (4.6)	39 (22.5)	
Educational level, *n* (%)				**< 0.001**
Lower primary	462 (52.0)	399 (55.8)	63 (36.4)	
Upper primary	242 (27.3)	179 (25.0)	63 (36.4)	
Lower secondary	82 (9.2)	67 (9.4)	15 (8.7)	
Upper secondary	40 (4.5)	25 (3.5)	15 (8.7)	
Tertiary	42 (4.7)	31 (4.3)	11 (6.4)	
No education	20 (2.3)	14 (2.0)	6 (3.5)	
Occupation, *n* (%)				**< 0.001**
Used	77 (8.7)	57 (8.0)	20 (11.6)	
Farming	591 (66.6)	493 (69.0)	98 (56.6)	
Housewife	153 (17.2)	107 (15.0)	46 (26.6)	
Tailor	67 (7.5)	58 (8.1)	9 (5.2)	
Religion, *n* (%)				0.714
Anglican/Protestant	236 (26.6)	189 (26.4)	47 (27.2)	
Catholic	549 (61.8)	440 (61.5)	109 (63.0)	
Muslim	6 (0.7)	5 (0.7)	1 (0.6)	
Other	94 (10.6)	80 (11.2)	14 (8.1)	
Missing	3 (0.3)	1 (0.1)	2 (1.2)	
Marital status, *n* (%)				0.879
Divorced	10 (1.1)	8 (1.1)	2 (1.2)	
Married	830 (93.5)	667 (93.3)	163 (94.2)	
Never married	48 (5.4)	40 (5.6)	8 (4.6)	
Ever diagnosed with COVID-19, *n* (%)	4 (0.5)	4 (0.6)	0 (0)	0.723
Missing	1 (0.1)	1 (0.1)	0 (0)	
Received vaccination against COVID-19, *n* (%)	817 (92.0)	663 (92.7)	154 (89.0)	0.128
Missing	1 (0.1)	1 (0.1)	0 (0)	
Vaccine doses, *n* (%)				0.138
More than two doses	119 (13.4)	89 (12.4)	30 (17.3)	
Two doses	353 (39.8)	293 (41.0)	60 (34.7)	
One dose	345 (38.9)	281 (39.3)	64 (37.0)	
Unvaccinated	71 (8.0)	52 (7.3)	19 (11.0)	
Belief that COVID is more dangerous than malaria, *n* (%)	593 (66.8)	491 (68.7)	102 (59.0)	**0.0199**
Missing	4 (0.5)	3 (0.4)	1 (0.6)	
Good knowledge about malaria, *n* (%)	316 (35.6)	236 (33.0)	80 (46.2)	**0.0015**

IQR = interquartile range.

* Chi-squared test or *t* test, as appropriate. Bold *P* values represent statistically significant variables.

Ninety-two percent (*n* = 817) of the women reported being vaccinated against COVID-19, but the rate of vaccination with more than two doses was 13.4% (*n* = 119). Only four women (0.05%), all belonging to the “poor COVID knowledge group,” reported having been diagnosed with SARS-CoV2 infection in the past. Most women (66.8%, *n* = 593) believed that COVID-19 is more dangerous than malaria.

**Figure 1.**
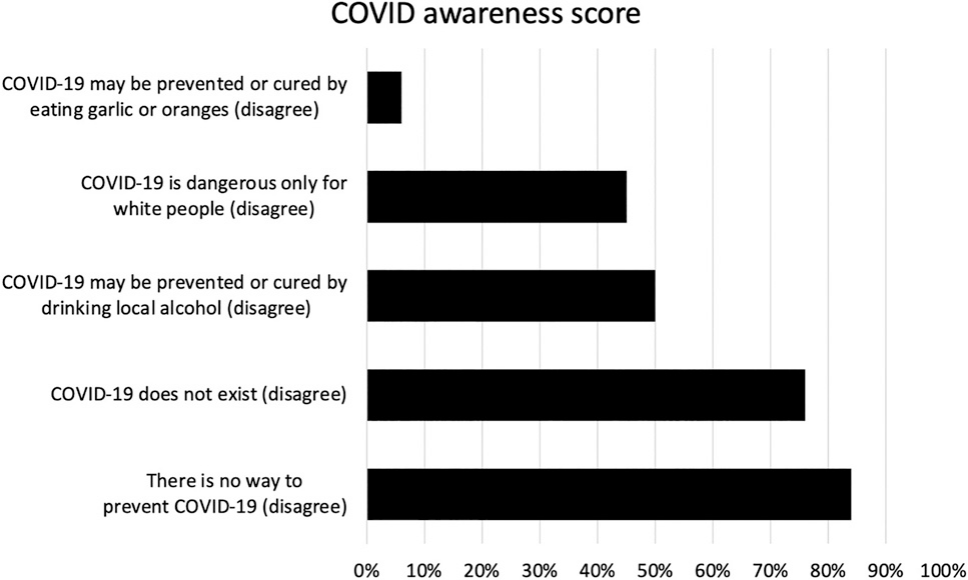
In the multivariate analysis (see Table 2), variables associated with high COVID awareness were as follows: presenting at antenatal care visit in Atipe health center (adjusted odds ratio [aOR] 8.1, 95% CI: 4.1–16.48), having attained at least upper secondary school (aOR 3.05, 95% CI: 1.36–6.7), and having good knowledge about malaria (aOR 1.76, 95% CI: 1.21–2.56).

Figure 1 shows the results of the COVID-19 awareness score. Overall, 173 women scored at least 4 and were identified as having good knowledge about COVID-19. At this regard, 93.8% (*n* = 833) of the women believed that COVID-19 might be prevented by eating garlic or mangoes, 50% (*n* = 444) by drinking a locally produced alcoholic beverage, and 55.2% (*n* = 490) thought that COVID-19 is dangerous only for White people.

**Table 2:** Multivariate logistic regression analysis

Variable	aOR	95% CI low	95% CI high	*P* value
Age	1.00	0.972	1.038	0.75
Presentig at Aboke HC for ANC visit	3.22	0.622	20.667	0.19
Presenting at Atipe HC for ANC visit	8.179	4.146	16.484	**< 0.001**
Highest level of education that you attained: upper secondary	3.052	1.359	6.708	**0.006**
Occupation: farming	0.828	0.414	1.718	0.603
Occupation: unemployed	0.821	0.369	1.854	0.632
Occupation: tailor	0.519	0.194	1.316	0.176
Belief that COVID is more dangerous than malaria	0.506	0.34	0.753	**< 0.001**
Good knowledge about malaria	1.761	1.211	2.56	**0.003**

ANC = antenatal care; aOR = adjusted odds ratio; HC = health center. Bold *P* values represent statistically significant variables.

## DISCUSSION

Despite their similar clinical presentation, both SARS-CoV2 and *P. falciparum* infection poses a substantial threat for pregnant women and their fetuses, who are at increased risk to progress toward pregnancy loss, severe disease, and death. The present study aimed to explore the awareness of COVID-19 among pregnant women living in a rural area in northern Uganda with a high burden of *P. falciparum* malaria.

In this study, we found a very poor level of COVID-19 awareness, with four out of five women scoring below the threshold of high awareness. This is consistent with findings from a recent systematic review and meta-analyses investigating COVID-19 knowledge among pregnant women conducted by Jahromi et al.,^16^ in which Uganda ranked as the country with the lowest level of awareness about SARS-CoV2 infection. Also, the present study confirms that COVID-19 awareness in Uganda was generally lower than the one found in other sub-Saharan African countries.^17^

In particular, in this study, most women believed in common misconceptions, such as that COVID-19 might be prevented by eating garlic or mangoes or by drinking *waragi*: a locally produced, hard alcohol distilled spirit. Worryingly, this fallacy was identified also in a survey conducted by the United Nations High Commissioner for Refugees^18^ in Congo, South Sudan, Uganda, Rwanda, and Burundi in late 2020, thus underscoring the fact that some diffuse—and potentially harmful—misconceptions should be explicitly addressed by public health interventions.^19^

Furthermore, more than half of the pregnant women included in our sample thought that COVID-19 is dangerous only for White people. In fact, the belief that COVID-19 is dangerous only for White, rich people appears to be a widespread belief in sub-Saharan Africa.^20^ This is consistent with the finding that, compared with high-income countries, the estimated infection-fatality ratio of SARS-CoV2 infection was significantly lower in Sub-Saharan African countries, even when controlling for age, vaccination status, clinical covariates, and health system performances.^21^ Several hypotheses have been proposed to explain the lower than anticipated burden of severe COVID-19 in sub-Saharan Africa, including age structures, the hygiene hypothesis,^22^ microbiome diversity,^23^ and previous malaria exposure.^24^

Another peculiar finding of this study is that two-thirds of the recruited women believed that COVID is more dangerous than malaria. This is interesting because it goes in contrast with the disease burden experienced by the pour population, both in terms of COVID-19 and malaria, because only four women reported to have ever been diagnosed with COVID-19. Even though SARS-CoV2 infection rate was likely higher,^25^ this finding deserves consideration because is a proxy for COVID-induced medical attention seeking. Also, as part of a broader ongoing cohort study,^2^ *P. falciparum* infection rate is under investigation in the same population, and preliminary analysis suggest that blood smear positivity rate is as high as 18% (unpublished data). In Uganda, malaria is the second leading cause of all-age death and disability^26^ and the first nonobstetric cause of death among hospitalized pregnant women.^27^ For these reasons, to implement the delivery of services to pregnant women, a collaboration between local and nongovernmental organizations was of a great importance together with the role of community health workers in the promotion of the use of antenatal clinic services.^28^ In the present study, a correct perception of the relative danger posed by COVID-19 compared with malaria correlates with an overall high awareness of COVID-19 at multivariable analysis.

Despite the high diffusion of COVID misconceptions, overall adherence to the vaccination against SARS-CoV2 was very high. These data corroborate the findings that in low- and middle-income countries, vaccine acceptance is generally high, sometimes even higher than that reported in most high-income countries.^29^

At multivariate analysis, other factors that were identified to be associated with good COVID awareness were higher educational level, good knowledge about malaria, and visiting the ANC clinic located in Atipe HC. During the pandemic, the same educational intervention was carried out in all three study sites, but by different teams; therefore, this finding suggests that healthcare officers may play a crucial role in disseminating accurate information and promoting awareness among pregnant women. Further studies are required to assess the impact of standardized educational strategies in improving disease awareness and promote prevention.

This study has several limitations that should be acknowledged. First, it was conducted in a specific region of rural Uganda, and the sample may not be representative of the entire country or other contexts. However, sample size was high, and our sample should be representative of the population of pregnant women living in rural areas of Northern Uganda. Second, the study relied on self-reported data, which may be subject to social desirability bias. However, to minimize this bias, research assistants working in ANC clinic were trained to create a safe space for pregnant women, encouraging them to be sincere and not to feel judged. Third, the 5-point score used as study endpoint was highly context- and disease-specific, which limits its reproducibility. Fourth, we did not directly assess preventive behaviors.

## CONCLUSIONS

In conclusion, this study provides valuable insights into the awareness of COVID-19 among pregnant women in a malaria hyperendemic region of rural Uganda. The findings highlight the presence of misconceptions and knowledge gaps that need to be addressed through targeted health education interventions. It is mandatory to implement plans and strategies for malaria and COVID-19 prevention with proper information and interventions, placing importance on the collaboration between clinicians and maternal health experts. Furthermore, a monitoring program of education with an appropriate update over time is essential. Strengthening health education within healthcare facilities, promoting literacy and education, and integrating disease-specific knowledge can enhance the overall understanding of both malaria and COVID-19. In *P. falciparum* hyperendemic areas, community-level malaria awareness among pregnant women might guide educational interventions during future pandemics.

## Financial Disclosure

This research was partially supported by EU funding within the NextGenerationEU-MUR PNRR Extended Partnership initiative on Emerging Infectious Diseases (Project no PE00000007, INF-ACT).
